# Acknowledgement of manuscript reviewers 2015

**DOI:** 10.1186/s13052-016-0232-0

**Published:** 2016-02-26

**Authors:** Sergio Bernasconi, Carlo Caffarelli, Francesca Santamaria

**Affiliations:** University of Parma, Parma, Italy; Federico II University, Napoli, Italy

## Abstract

The editors of *Italian Journal of Pediatrics* would like to thank all our reviewers who have contributed to the journal in Volume 41 (2015).

**Ahmad Abolyosr**

Egypt

**Gabriel Agboado**

UK

**Mehmet Agilli**

Turkey

**Carlo Agostoni**

Italy

**Khalid Alansari**

Qatar

**Gosta Alfven**

Norway

**Charles Algert**

Australia

**Jose Alonso Fernandez**

Suriname

**Sergio Amarri**

Italy

**Generoso Andria**

Italy

**Percesepe Antonio**

Italy

**Maurizio Aricò**

Italy

**Nur Arslan**

Turkey

**M Awazu**

Japan

**Monica Badve**

Australia

**Tahmina Banu**

Bangladesh

**Egidio Barbi**

Italy

**Donald Barker**

USA

**Giovanni Barone**

Italy

**Romain Basmaci**

France

**Luisa Bay**

Italy

**Carlo Bellieni**

Italy

**Marcello Bergamini**

Italy

**Roberto Berni Canani**

Italy

**Vishnu Bhat**

India

**Giacomo Biasucci**

Italy

**Paolo Biban**

Italy

**G Biglino**

UK

**Andrea Biondi**

Italy

**Hurrem Bodur**

Turkey

**Antonio Boldrini**

Italy

**Roberta Bombardieri**

Italy

**Gianni Bona**

Italy

**Marco Bonvicini**

Italy

**Paolo Bottau**

Italy

**Cesare Braggion**

Italy

**Francesco Brancati**

Italy

**Davide Brancato**

Italy

**Carmela Bravaccio**

Italy

**Silvia Bressan**

Italy

**Jeffrey Bridge**

USA

**Elizabeth Brownell**

USA

**N Brunetti Pierri**

Italy

**Nicola Brunetti-Pierri**

Italy

**Oliviero Bruni**

Italy

**Yenan Bryceson**

Sweden

**Giuseppe Buonocore**

Italy

**Jeremy Burton**

Canada

**V Caldarelli**

Italy

**Mario Canciani**

Italy

**Marco Cappa**

Italy

**Manuela Caruso**

Italy

**Alessandra Cassio**

Italy

**Francesco Castelli**

Italy

**Marco Castori**

Italy

**Marco Cattalini**

Italy

**Claudio Cavalli**

Italy

**Simone Cesaro**

Italy

**Enrico Chiappa**

Italy

**Francesco Chiarelli**

Italy

**Gaetano Chirico**

Italy

**C F Christiansen**

Denmark

**Rene Chun**

USA

**Ondrej Cinek**

Czech Republic

**Emilia Cirillo**

Italy

**Paola Cogo**

Italy

**Pierluigi Colonna**

Italy

**S Contini**

Italy

**L Copelovitch**

USA

**Samuele Cortese**

UK

**Carlo Dani**

Italy

**Enza D'Auria**

Italy

**Omar Dawood**

Malaysia

**Maria Pia De Carolis**

Italy

**Valentina De Cosmi**

Italy

**Cosimo De Filippis**

Italy

**Gianpaolo De Filippo**

France

**Costantino De Giacomo**

Italy

**Maurizio De Martino**

Italy

**Luisa De Sanctis**

Italy

**Ennio Del Giudice**

Italy

**Joep Derikx**

Netherlands

**Binyam Desta**

Ethiopia

**A Dhakal**

Nepal

**Salvatore Di Maio**

Italy

**Michael Di Maria**

USA

**Ener Dinleyici**

Turkey

**John Dodge**

UK

**Icilio Dodi**

Italy

**A Durward**

UK

**C Duurland**

UK

**Ahmed Elhassanin**

Egypt

**Maurizio Elia**

Italy

**Bertazzoni Minelli Elisa**

Italy

**M El-Naggari**

Oman

**Leonardo Emberti Gialloreti**

Italy

**R Epaud**

France

**Gabriel Escobar**

USA

**Susanna Esposito**

Italy

**Carla Evans**

Australia

**Fernanda Falcini**

Italy

**Jessica Farebrother**

Switzerland

**Christiana Farkhou**

USA

**Pietro Ferrara**

Italy

**Ruth Festl**

Germany

**A Fierabracci**

Italy

**Michael Fietz**

Australia

**Alberto Flores D'Arcais**

Italy

**Fabrizio Franceschini**

Italy

**A Franco**

USA

**Adriana Franzese**

Italy

**Aaron Friedman**

USA

**Claudia Fsadni**

Malta

**Luigi Gagliardi**

Italy

**Cinzia Galasso**

Italy

**Paolo Gancia**

Italy

**Luis Garcia-Marcos**

Spain

**Roberto Gastaldi**

Italy

**Ying Ge**

China

**V Gerodimos**

Greece

**Majid Ghayour-Mobarhan**

Iran

**Gobbi Giuseppe**

Italy

**M Gonzalez-Gay**

Spain

**Glenn Gourley**

USA

**Salvatore Grosso**

Italy

**Graziano Grugni**

Italy

**Maa Guimaraes**

Brazil

**Smail Hadj-Rabia**

France

**Mayumi Hangai**

Japan

**Yukihiro Hasegawa**

Japan

**Richard Hegele**

Canada

**Kota Hirai**

Japan

**Elisabeth Hodson**

Australia

**Michael Holick**

USA

**S M Holland**

USA

**Katharina Holstein**

Germany

**Teuta Hoxha**

Albania

**R Iorio**

Italy

**Salvatore Italia**

Netherlands

**Lorenzo Iughetti**

Italy

**Lucky Jain**

USA

**Michael Kaplan**

Israel

**Hiroyuki Karibe**

Japan

**Ryou Kawamata**

Japan

**Paul Kelly**

UK

**Myungshin Kim**

Korea, South

**C M Kneepkens**

Netherlands

**Kaija-Leena Kolho**

Finland

**Nageswara Koneti**

India

**Andreas Konstantopoulos**

Greece

**Ho-Chang Kuo**

Taiwan

**Taiwo Ladapo**

Nigeria

**Jos Latour**

UK

**Eric Legius**

Belgium

**Salvatore Leonardi**

Italy

**Chien-Chang Liao**

Taiwan

**Thomas Liehr**

Germany

**Mario Lima**

Italy

**Luca Lo Nigro**

Italy

**Enrico Lombardi**

Italy

**Fortunato Lombardo**

Italy

**Fortunato Lonardo**

Italy

**M Lopes**

Brazil

**Riccardo Lubrano**

Italy

**Rita Luciano**

Italy

**Vincenzina Lucidi**

Italy

**M Ludwig**

Germany

**Maria Maddalena Lupica**

Italy

**Francesco Macrì**

Italy

**Giuseppe Maggiore**

Italy

**Akash Malik**

India

**Maria Marcovecchio**

Italy

**Alberto Martelli**

Italy

**Massimo Martinelli**

Italy

**Saurabh Maru**

India

**Gianfranco Mazzarella**

Italy

**Donna McDonald-McGinn**

USA

**Sheena Mcgowan**

UK

**H Meissner**

USA

**Jean-Christophe Mercier**

France

**Jean-Francois Michel**

Luxembourg

**F Midulla**

Italy

**Erasmo Miele**

Italy

**Roberto Miniero**

Italy

**Giuseppe Miragliotta**

Italy

**Th Mogensen**

Denmark

**Lorenzo Morelli**

Italy

**Essam Eldin Moursy**

Egypt

**Enza Mozzillo**

Italy

**Majigo Mtebe**

Tanzania

**Florida Muro**

Tanzania

**Philip Murray**

UK

**Michele Mussap**

Italy

**Iria Neri**

Italy

**Daniel Ng**

Hong Kong

**Antonio Niccoli**

Italy

**Rozina Nuruddin**

Pakistan

**Alexander Opotowsky**

USA

**Verity Pacey**

Australia

**Retno Palupi-Baroto**

Indonesia

**Mohan Pammi**

USA

**Claudia Pansieri**

Italy

**Piermichele Paolillo**

Italy

**Pasquale Parisi**

Italy

**Antonia Parmeggiani**

Italy

**Stefano Parmigiani**

Italy

**Niccolò Parri**

Italy

**Robert Partridge**

USA

**Nehal Patel**

India

**Annalisa Patrizi**

Italy

**M Pavone**

Italy

**Piero Pavone**

Italy

**Giorgio Perilongo**

Italy

**Diego Peroni**

Italy

**Laura Perrone**

Italy

**Andrea Pession**

Italy

**Giorgio Piacentini**

Italy

**Marco Piastra**

Italy

**Claudio Pignata**

Italy

**A Pini Prato**

Italy

**Francesco Pisani**

Italy

**Alessandro Plebani**

Italy

**Maria Cristina Porfirio**

Italy

**Carlotta Povesi Dascola**

Italy

**Akila Prashant**

India

**Simone Pratesi**

Italy

**Nicola Principi**

Italy

**Ninlapa Pruksanusak**

Thailand

**Hildegard Przyrembel**

Germany

**B Pyun**

Korea, Republic of

**Nashmia Qamar**

USA

**Paolo Quitadamo**

Italy

**Giorgio Radetti**

Italy

**L Raffini**

USA

**Valeria Raia**

Italy

**G Redding**

USA

**Judy Rees**

USA

**Florence Remy**

France

**Patrizia Restani**

Italy

**J Rettig**

Germany

**Giampaolo Ricci**

Italy

**Joan Robinson**

Canada

**Alan Rogol**

USA

**Costantino Romagnoli**

Italy

**Claudio Romano**

Italy

**Barbara Rombi**

Italy

**Massimiliano Rossi**

France

**Giovanna Russo**

Italy

**Maria Russo**

Italy

**Giuseppe Saggese**

Italy

**Ravi Sahu**

USA

**Gemma Saint**

UK

**Mariacarolina Salerno**

Italy

**D Salvatore**

Italy

**Lamiya Samad**

UK

**Anahita Sanaei Dashti**

Iran

**Judith Sanchez-Manubens**

Spain

**Ion Sandu**

Romania

**Maria Santagati**

Italy

**Francesco Savino**

Italy

**Andrea Scaramuzza**

Italy

**Ms Shin**

Korea, Republic of

**Ana Simoes E Silva**

Brazil

**Gabriele Simonini**

Italy

**Sunit Singhi**

India

**Deborah Snijders**

Netherlands

**M Somaschini**

Italy

**Anna Rosa Soresina**

Italy

**Alberto Spalice**

Italy

**Ivonne Sponzilli**

Italy

**Alex Staffler**

Italy

**Annamaria Staiano**

Italy

**Paul Steinbok**

Canada

**Maria Street**

Italy

**Paolo Tagliabue**

Italy

**Giovanni Tarantino**

Italy

**Ali Taylan**

Turkey

**Bertrand Tchana**

Italy

**Luigi Terracciano**

Italy

**Anna Maria Testi**

Italy

**Luigi Titomanlio**

France

**Shunji Tomatsu**

Japan

**Tuomo Tompuri**

Finland

**Nuria Torner**

Spain

**Stefania Toselli**

Italy

**E Trayer**

Hungary

**Daniele Trevisanuto**

Italy

**Birger Trollfors**

Sweden

**Boldtsetseg Tserenpuntsag**

USA

**Tuba Tuncel**

Turkey

**Ja Tur**

Spain

**Daniel Umpierre**

Brazil

**Erbil Unsal**

Turkey

**Eleonora Uphoff**

UK

**Pietro Vajro**

Italy

**Enrico Valletta**

Italy

**Wendy Van Lippevelde**

Belgium

**Gijs Van Well**

Netherlands

**Job Van Woensel**

Netherlands

**N Vatanavicharn**

Thailand

**Maria Carmela Verga**

Italy

**Alberto Verrotti**

Italy

**Paolo Villani**

Italy

**Vincenzo Vincenti**

Italy

**Anju Virmani**

India

**Nj Vo**

USA

**Malgorzata Wasniewska**

Italy

**Tara Wenger**

USA

**Ephraim Winocur**

Israel

**Huiyun Xiang**

USA

**Enrico Zecca**

Italy

**Qingguo Zhao**

China

**Ralph Ziegler**

Germany

**G Zuccotti**

Italy

**Gian Vincenzo Zuccotti**

Italy

**Antonio Zuppa**

Italy

